# Water Activity Prediction in Sugar and Polyol Systems Using Theoretical Molecular Descriptors

**DOI:** 10.3390/ijms222011044

**Published:** 2021-10-13

**Authors:** Antonio Zuorro

**Affiliations:** Department of Chemical Engineering, Materials and Environment, Sapienza University, 00185 Rome, Italy; antonio.zuorro@uniroma1.it

**Keywords:** water activity, sugars, polyols, Norrish model, molecular descriptors, information theory

## Abstract

Water activity is a key factor in the development of pharmaceutical, cosmetic, and food products. In aqueous solutions of nonelectrolytes, the Norrish model provides a simple and effective way to evaluate this quantity. However, it contains a parameter, known as the Norrish constant, that must be estimated from experimental data. In this study, a new strategy is proposed for the prediction of water activity in the absence of experimental information, based on the use of theoretical molecular descriptors for characterizing the effects of a solute. This approach was applied to the evaluation of water activity in the presence of sugars (glucose, fructose, xylose, sucrose) and polyols (sorbitol, xylitol, glycerol, erythritol). The use of two descriptors related to the constitutional and connectivity properties of the solutes was first investigated. Subsequently, a new theoretical descriptor, named the global information index (*G*), was developed. By using this index, the water activity curves in the binary systems were reconstructed. The positive results obtained support the proposed strategy, as well as the possibility of including, in a single information index, the main molecular features of a solute that determine its effects on water activity.

## 1. Introduction

Water activity is one of the most important factors influencing the quality and stability of food, cosmetic, and pharmaceutical products [1,2,3]. Most of the studies on water activity have been carried out on food products, since this quantity has a significant effect on microbial stability, shelf life, and organoleptic characteristics [4].

Formal recognition of the importance of water activity dates back to the early 1950s, when Scott conducted a pioneering study showing that microbial growth and toxin production in food products were dependent on the activity, not the content, of water [5].

Although water activity can be rigorously defined in thermodynamic terms [6], its molecular origin is still far from being understood, as evidenced by the existence of different and sometimes conflicting explanations [7]. The most popular is that of “free water”, according to which water activity reflects its availability as a solvent or reagent, which results from the interactions between water molecules [8]. Another explanation attributes its origin to the structuring or ordering of water molecules induced by a solute [9]. In particular, a solute can behave as a “structure maker” or a “structure breaker”, depending on its ability to enhance or weaken the hydrogen-bonded water network. A further interpretation is based on the concepts of solute clustering and hydration number, that is, the number of water molecules close to the solute [10].

Despite the molecular significance of water activity remaining somewhat elusive, the importance of evaluating this quantity for the systems of interest is evident. For this purpose, many empirical and semi-empirical models have been developed [8,11,12]. One of the most used is the Norrish model, which provides a good compromise between accuracy and simplicity [13]. For a single-solute system, this model contains only one parameter, known as the Norrish constant, which can be easily determined from experimental data. However, in many situations, it may be necessary to estimate water activity in the absence of experimental information.

Molecular descriptors are numeric quantities associated with some structural feature or property of a molecule [14]. The use of these descriptors for property prediction has its basis in the principle of similarity, according to which similar molecular structures have similar chemical properties, just as different molecular structures have different chemical properties. Over the years, thousands of molecular descriptors have been used to predict the properties of various substances [15]. They can be classified into the following main categories [16,17]: constitutional; topological; geometrical; and quantum–chemical descriptors. Constitutional descriptors of a compound are based on the number and types of atoms, bonds, rings, etc., in its molecule. Topological descriptors are related to the two-dimensional structure of the molecule, which is regarded as a graph, with vertices representing atoms and edges representing bonds. Geometrical descriptors are derived from the three-dimensional structure of the molecule and consider different molecular features, such as molecular volume, total surface area, and solvent-accessible surface area. Finally, quantum–chemical descriptors are obtained from quantum–mechanical calculations aimed at characterizing the electronic properties of the molecule.

The aim of this study was to investigate whether the activity of water in the presence of polyols or sugars could be predicted using some theoretical descriptors of the solutes. Attention is focused on sugars and polyols, since they are widely used as protein stabilizers or cryoprotectants [18,19], as well as for the control of microbial growth in food products [20]. The results obtained showed that the Norrish constant can be well described by two theoretical indices related to the constitutional and connectivity properties of the solutes: the information index on atomic composition and the first Zagreb index. They were selected among the thousands of currently available descriptors due to their ease of computation and well-known correlation with important molecular and physico–chemical properties. In addition, a new theoretical descriptor was developed by combining the above indices. To the author’s knowledge, this is the first attempt to use an approach based on theoretical molecular descriptors to predict water activity.

## 2. Results and Discussion

As a first step to investigate the potential of molecular descriptors to predict the activity of water in the presence of polyols or sugars, the experimental activity data were correlated by the Norrish model. The solutes were then characterized using different descriptors, and their ability to predict the Norrish constant was evaluated. Finally, a new descriptor combining information on atomic composition and molecular connectivity was developed and used to reconstruct the water activity curves in the systems studied.

### 2.1. Correlation of Water Activity Data

For a single-solute system, the Norrish model provides the following expression for the dependence of water activity (*a_w_*) on composition:

(1) $$a_{w}=x_{w}e^{k_{N}x_{s}^{2}},$$

where *k_N_* is the Norrish constant, *x_w_* is the mole fraction of water, and *x_s_* is the mole fraction of the solute. Equation (1) can be derived rigorously from the Kirkwood–Buff theory of solutions as shown in Appendix A, where the thermodynamic meaning of *k_N_* can also be deduced.

The correlation of water activity data by Equation (1) was performed by minimization of the following objective function:

(2) $$\Phi \left(k_{N}\right)=\overset{n}{\underset{i=i}{\sum }}{\left(a_{wi,exp}-a_{wi,calc}\right)}^{2},$$

where *n* is the number of points of each data set, and the subscripts *exp* and *calc* indicate experimental and calculated values. Since the latter depend on the Norrish constant, it follows that Φ = *f*(*k_N_*). The results of the estimation procedure are summarized in Table 1, where the mean absolute error, defined as:

(3) $$\varepsilon =\frac{1}{n}\left|a_{wi,exp}-a_{wi,calc}\right|,$$

is also reported. The excellent agreement between experimental and calculated results (2.71 × 10^−4^ ≤ ε ≤ 1.35 × 10^−3^) clearly attests the suitability of the Norrish model to describe the activity of water in the investigated systems.

### 2.2. Use of Molecular Descriptors for the Prediction of the Norrish Constant

Different molecular descriptors were examined for their ability to predict the Norrish constant, with a focus on the classes of constitutional and topological indices.

Constitutional indices are zero-dimensional descriptors. They are the simplest and most used descriptors of a molecule, since they relate to easily determinable molecular features, such as the type of atoms, functional groups, bonds, or number of rings. In this study, the total information index on atomic composition (*I_AC_*) was selected to describe the constitutional properties of the solutes. *I_AC_* provides information about the type of atoms present in the molecule. This was the first theoretical information index introduced by Dancoff and Quastler in the early 1950s [21]. Since then, an increasing number of studies has revealed the importance of this and other composition-related descriptors in the development of structure–property relationships [22].

Topological indices are two-dimensional descriptors derived from the topological representation of a molecule. In recent decades, molecular topology has emerged as a powerful approach to evaluate structure–activity relationships [23], especially in the fields of pharmacology and toxicology [24,25]. According to this approach, molecular structures are described in terms of the mathematical properties of their associated graphs. For organic compounds, H-depleted graphs (i.e., graphs not including hydrogen atoms) are usually considered, due to the supposed limited contribution of these atoms to molecular connectivity. In this study, the first Zagreb index (*Z*_1_) was selected as a measure of molecular connectivity. *Z*_1_ belongs to the class of the Kier–Hall connectivity indices, and is one of the oldest and most studied topological descriptors [26]. It is related to the concept of vertex valency, and therefore characterizes the degree of atomic branching in the molecule.

With respect to the solutes examined here, it is interesting to consider that glucose and fructose, being isomers, have the same chemical formula. Accordingly, they are characterized by the same *I_AC_* value. However, their molecular connectivity shows some differences, which are reflected in the different values of *Z*_1_. In other words, contrary to the information index on atomic composition, the Zagreb index allows for discrimination between the two isomers. This is an important point to highlight since, as can be seen from the experimental activity data (Tables S1 and S2) and the estimated Norrish constants (Table 1), the two solutes affect the activity of water differently.

To express the dependence of the Norrish constant on the selected descriptors, two empirical models were initially used: the linear model and the exponential model. They are described, respectively, by Equations (4) and (5):

(4) $$k_{N}=a_{11}+a_{12}I_{AC}+a_{13}Z_{1},$$

(5) $$k_{N}=a_{21}I_{AC}{}^{a_{22}}Z_{1}{}^{a_{23}}.$$

Both models contain three parameters, which were estimated by the least-squares procedure, yielding the results presented in Table 2. The quality of correlation was evaluated by calculating the following quantity:

(6) $$\varphi =\frac{\Theta }{n-p},$$

where Θ is the sum of squared errors between experimental and calculated Norrish constants, *n* is the number of data points, and *p* is the number of model parameters. It can be noticed that *ϕ* represents an estimate of the model variance, and can therefore be related to the predictive accuracy of the model.

As a further step in the search of a relationship between *k_N_* and the molecular features of the solutes, a new descriptor, named the global information index (*G*), was developed by combining the information concerning atomic composition and molecular connectivity. It was defined as:

(7) $$G=I_{AC}+Z_{1}.$$

A similar approach, based on the consideration that indices describing specific characteristics of the molecule can be combined with one another, was used to describe the behavior of other systems of different complexities. Some examples are provided by the Bertz index, the Dosmorov index, and the topological superindex, which are described in [27].

The *G* index still allows for discrimination between glucose and fructose, being that their connectivity contributions are different. Moreover, the data were correlated using the linear (Equation (8)) and the exponential (Equation (9)) models:

(8) $$k_{N}=b_{11}+b_{12}G,$$

(9) $$k_{N}=b_{21}G^{b_{22}}.$$

In addition, the following second-order polynomial model was considered:

(10) $$k_{N}=b_{31}G+b_{32}G^{2}.$$

Each of the above models contains two parameters, against the three of the two-descriptor models. Limiting the number of parameters in a model is desirable for reducing the risk of overfitting, especially when empirical models are considered [28]. In this regard, it may be useful to observe that the *ϕ* quantity accounts for the number of parameters in the model, allowing for a comparison of models differing in the number of parameters.

As is evident from Table 2, the single-index models improved the quality of correlation, compared to their two-descriptor counterparts. Among the single-index models, the lowest *ϕ* value (5.17 × 10^−2^) was achieved with the polynomial model (Equation (10)); therefore, it was selected as the most appropriate model for evaluating water activity.

**Figure 1:**
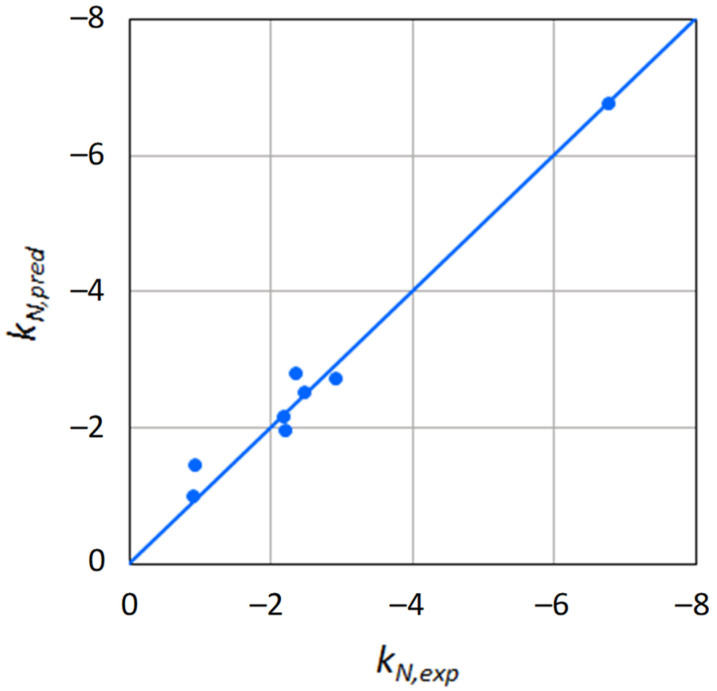
Comparison between experimental (*k_N,exp_*) and predicted (*k_N,pred_*) Norrish constants.

**Figure 2:**
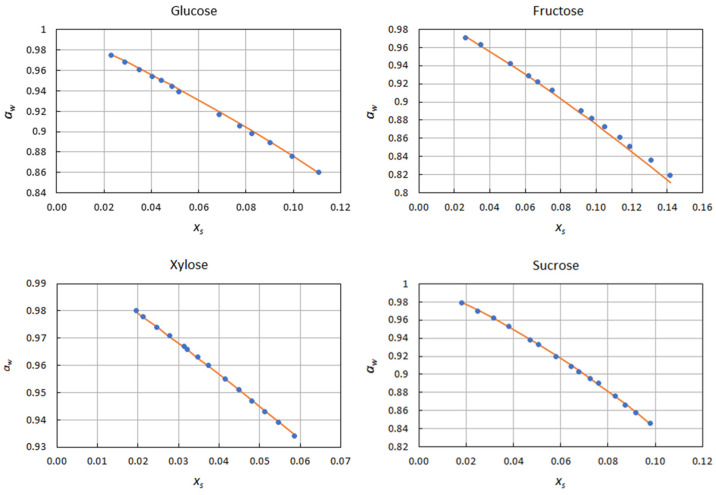
Water activity curves in the presence of sugars predicted by Equation (10), and comparison with the experimental data points.

**Figure 3:**
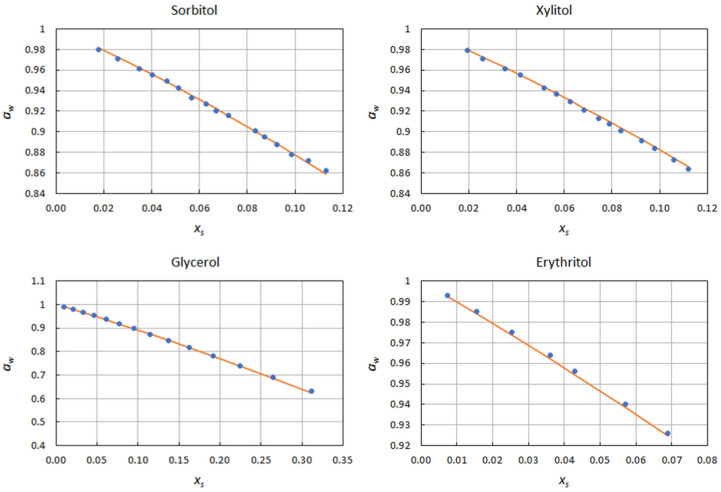
Water activity curves in the presence of polyols predicted by Equation (10), and comparison with the experimental data points.

Figure 1 shows a comparison between experimental (i.e., determined from the experimental activity data) and predicted Norrish constants. The latter were used to reconstruct the activity curves, and are displayed in Figure 2 and Figure 3, together with the experimental data points. The excellent agreement between predicted and experimental water activities clearly demonstrates the model’s ability to describe the effects of solutes on water activity. This was true for both sugars and polyols.

**Figure 4:**
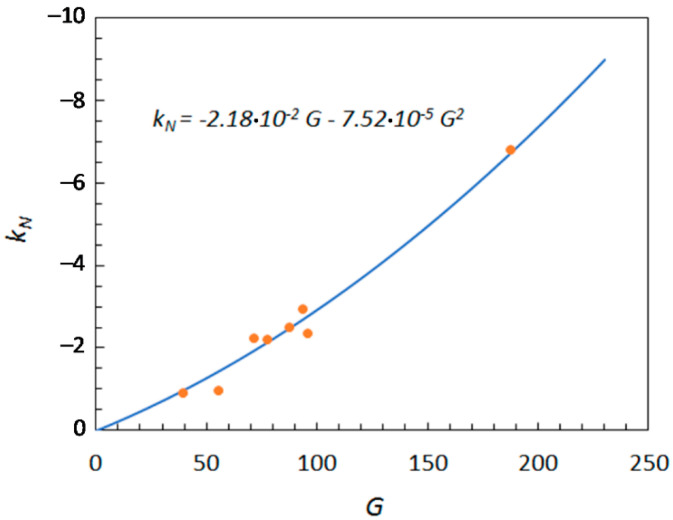
Effect of the global information index (*G*) on the Norrish constant (*k_N,_*), according to the second-order polynomial model (Equation (10)). Data points represent the experimental Norrish constant values.

**Figure 5:**
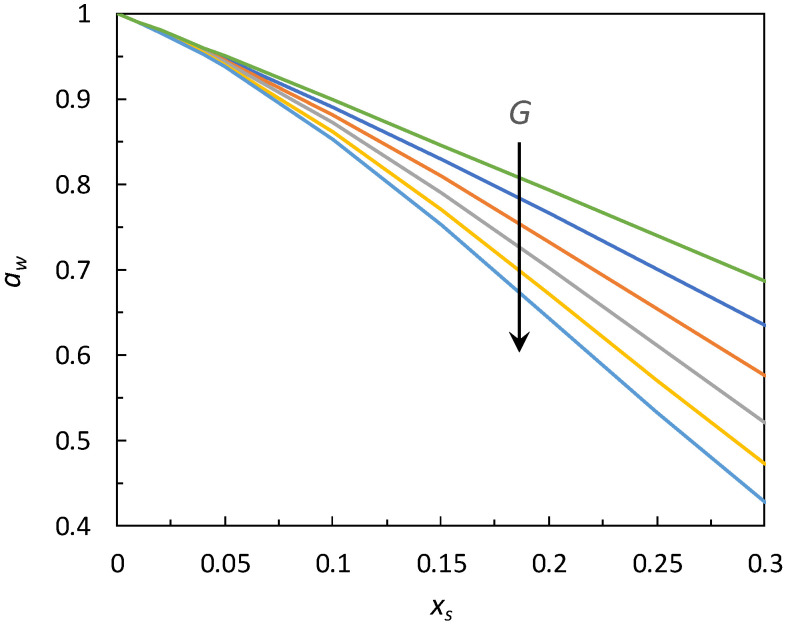
Effect of the global information index (*G* = 10, 50, 100, 150, 200, 250, in the direction indicated by the arrow) on water activity curves.

Figure 4 shows the dependence of *k_N_* on *G*, according to Equation (10), while the predicted effects of *G* on water activity are displayed in Figure 5. It can be seen that, for small solute additions, approximately below *x_s_* = 0.025–0.03, the activity curves are practically unaffected by the *G* value, that is, by the solute nature. At higher solute concentrations, the effects of *G* become more pronounced, and the curves tend to progressively move away from each other.

The ability of the *G* index to describe the effects of a solute on water activity reflects the dependence of water activity on the compositional and connectivity features of the solute molecule.

In a study on the effects of nonelectrolyte solutes, including sugars and polyols, on water activity, Chirife et al. [29] found that these compounds caused a decrease in water activity, and that their activity-lowering ability was correlated with the number of hydroxyl groups in the molecule. In particular, the Norrish constant increased almost linearly with the number of hydroxyl groups. However, as outlined by the above authors and in later studies [30,31,32], not all hydroxyl-containing compounds fitted the same correlation, suggesting that, in addition to the number of OH-groups, other molecular characteristics, such as their orientation and possible steric constraints, might be involved.

In the liquid state, water molecules are associated by hydrogen bonding, and form a structured dynamic network [33]. The formation of this network is made possible by the tetrahedral structure of water, in which oxygen is located at the center of the tetrahedron, while the hydrogen atoms and the two oxygen lone pairs are positioned at the vertices. As a result, each water molecule is hydrogen-bonded to four other molecules. Water can act as both a hydrogen bond donor and acceptor, which has important implications for the properties of aqueous solutions [34]. Solutes containing hydroxyl groups can form hydrogen bonds with water, and therefore they can perturb the structure and dynamics of the water network. Depending on the relative contribution of solute–water, solute–solute and water–water interactions, the network can be strengthened or weakened. Solutes causing a reinforcement of the water network are called “structure makers”. This is the case, for example, of sucrose [35]. However, the balance of intermolecular interactions is also affected by temperature and solute concentration, meaning that the same solute can have different effects at different concentrations [33,36].

A molecular dynamics study investigated the effects of polyols, differing in both the number of hydroxyl groups and configuration, on water structuring [37]. All of them caused a perturbation of the hydrogen-bonded network structure, whose extent depended on the number of OH-groups and polyol conformation. Although there was only a limited effect on the total number of hydrogen bonds, compared to that exhibited in pure water, hydrogen bonds between water and polyol were weaker than those between water and water. This was likely due to steric hindrance effects and lower polarization of the hydrogen bond formed. Due to this unfavorable situation, the interaction between water molecules was strengthened.

In another molecular dynamics study on aqueous solutions of various osmolytes, including ethanol, glycerol, glucose, trehalose, and sorbitol, it was found that all of the studied osmolytes were well integrated into the hydrogen-bonded water network [38]. Furthermore, these compounds behaved as “hubs” in the network, with their degree of hydrogen bonding affecting the connectivity and other properties of the network.

It is, therefore, evident that the number and relative positions of hydroxyl groups in the solute molecule influence the structure of the water network. With regard to the global information index proposed here, it can be seen that its atomic-composition component is highly correlated (*R*^2^ = 0.928) with the number of hydroxyl groups in the solute molecule (Figure S1). Furthermore, the *G* index is also influenced by how the hydroxyl groups are arranged in the molecule, as their positions affect the connectivity component of the index. This is the case, for example, with glucose and fructose. Both sugars have five hydroxyl groups in their molecule; however, in glucose, they are all equatorially positioned, while in fructose, only three of the five are in this arrangement (Figure S2).

From the above, it can be concluded that the *G* index is able to capture the molecular features of the solutes that mostly affect the water network structure, and, thus, water activity [7,9,39]. Finally, a comparison of the performance of models containing *G* with those including *I_AC_* and *Z*_1_ suggests that there is no loss of information when the two indices are combined into a single one.

## 3. Methods

### 3.1. Studied Systems and Literature Data

This study was performed on binary aqueous solutions containing glucose, fructose, xylose, sucrose, sorbitol, xylitol, glycerol, and erythritol as solutes. The experimental data were taken from the literature [40,41], and consisted of water activity values at different solute concentrations (Tables S1–S8).

### 3.2. Molecular Descriptors

Molecular descriptors were calculated using the alvaDesc^®^ software tool (Alvascience Srl, Lecco, Italy), which allows for the evaluation of over 5000 types of descriptors based on the SMILES (Simplified Molecular Input Line Entry System) representation of the compound of interest. Some molecular properties of the investigated solutes relevant to the computation of descriptors are listed in Table 3.

The descriptors selected for the prediction of the Norrish constant were *I_AC_*, the information index on atomic composition, and *Z*_1_, the first Zagreb index.

*I_AC_* is related to the atomic composition of the molecule, including hydrogen atoms. It is calculated as:

(11) $$I_{AC}=A_{tot}\log_{2}A_{tot}-\sum_{j}A_{j}\log_{2}A_{j},$$

where *A_tot_* is the total number of atoms in the molecule and *Aj* is the number of atoms of type *j*. For example, in the case of glucose (C_6_H_12_O_6_), we have:

(12) $$I_{AC}=24\log_{2}24-[2\left(6\;\log_{2}6)+12\;\log_{2}12\right]=36.$$

**Figure 6:**
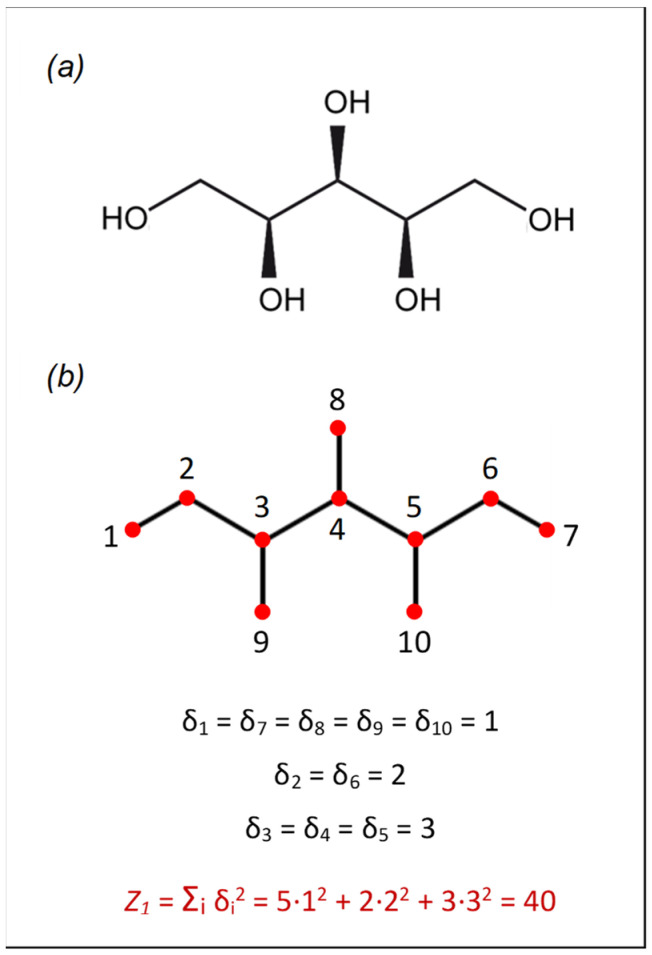
Calculation of the first Zagreb index (*Z*_1_) for xylitol: (**a**) chemical structure of xylitol; (**b**) hydrogen-depleted molecular graph. Graph vertices are numbered from 1 to 10. *δ_i_* is the vertex degree of vertex *i*.

*Z*_1_ is based on the concept of vertex degree, which is defined as the number of edges incident with a vertex of the molecular graph. It is calculated as:

(13) $$Z_{1}=\sum_{i=1}^{V}\delta_{i}^{2},$$

where *V* is the total number of vertices in the molecule and *δ_i_* is the vertex degree of vertex *i*. For molecules containing hydrogen atoms, the hydrogen-depleted graph is considered. An example of the calculation of *Z*_1_ for xylitol (C_5_H_12_O_5_) is shown in Figure 6.

The values of the information indices for the investigated solutes are reported in Table 4.

## 4. Conclusions

Water activity is widely recognized as a key factor in the development of food, cosmetics, and pharmaceutical products, due to its effects on product quality and shelf life. In this study, a new strategy for the prediction of water activity, based on the use of theoretical molecular descriptors, was proposed. An overall molecular descriptor is also developed, which takes into account the atomic composition and the connectivity of the solute molecule. The good results obtained support the possibility of including, in a single and easily calculable index, the main molecular features of a solute that determine its effect on water activity.

The proposed strategy can be used to estimate water activity in the absence of experimental data, or for a preliminary screening of activity-lowering solutes. Future studies should be directed at validating this approach on different classes of compounds and on multicomponent systems. Further molecular indices can also be developed, based on the mechanisms involved in the control of water activity. However, as suggested by the parsimony principle, the related mathematical models should be as simple as possible and contain the minimum number of parameters to make them reasonably accurate.

## Figures and Tables

**Table 1:** Estimated Norrish constant values (∆*x_s_*: experimental range of solute mole fractions; *k_N_*: Norrish constant; *Φ_min_*: minimum value of the objective function; *ε*: mean absolute error).

Solute	∆*x_s_*	*k_N_*	*Φ_min_*	*ε*
Glucose	0.023–0.111	−2.920	1.71 × 10^−5^	1.00 × 10^−3^
Fructose	0.026–0.142	−2.351	6.76 × 10^−6^	6.64 × 10^−4^
Xylose	0.020–0.059	−2.196	1.30 × 10^−6^	2.71 × 10^−4^
Sucrose	0.018–0.098	−6.777	6.87 × 10^−6^	5.85 × 10^−4^
Sorbitol	0.018–0.113	−2.494	4.18 × 10^−5^	1.35 × 10^−3^
Xylitol	0.020–0.112	−2.221	1.12 × 10^−6^	7.46 × 10^−4^
Glycerol	0.010–0.312	−0.908	1.65 × 10^−5^	9.70 × 10^−4^
Erythritol	0.007–0.069	−0.950	4.28 × 10^−6^	7.08 × 10^−4^

**Table 2:** Estimated parameters of the models for predicting the Norrish constant (*k_N_*) from information indices (*I_AC_*: information index on atomic composition; *Z*_1_: first Zagreb index; *G*: global information index; *Θ*: sum of squared errors; *ϕ:* statistical quantity defined by Equation (6)).

Model	Parameters	*Θ*	*Φ*
$k_{N}=a_{11}+a_{12}I_{AC}+a_{13}Z_{1}$	*a*_11_ = 1.333 *a*_12_ = −8.39 × 10^−2^ *a*_13_ = −1.87 × 10^−2^	4.15 × 10^−1^	8.29 × 10^−2^
$k_{N}=a_{21}I_{AC}{}^{a_{22}}Z_{1}{}^{a_{23}}$	*a*_21_ = −1.44 × 10^−2^ *a*_22_ = 1.026 *a*_23_ = 3.81 × 10^−1^	2.67 × 10^−1^	5.35 × 10^−2^
$k_{N}=b_{11}+b_{12}G$	*b*_11_ = 8.90 × 10^−1^ *b*_12_ = −3.98 × 10^−2^	4.98 × 10^−1^	8.29 × 10^−2^
$k_{N}=b_{21}G^{b_{22}}$	*b*_21_ = −7.65 × 10^−3^ *b*_22_ = 1.295	3.35 × 10^−1^	5.58 × 10^−2^
$k_{N}=b_{31}G+b_{32}G^{2}$	*b*_31_ = −2.18 × 10^−2^ *b*_32_ = −7.52 × 10^−5^	3.10 × 10^−1^	5.17 × 10^−2^

**Table 3:** Molecular properties of the solutes relevant to the calculation of theoretical descriptors (MW: molecular weight; NNHA: number of non-H atoms; NB: number of bonds; NR: number of rings).

Solute	Formula	MW	NNHA	NB	NR
Glucose	C_6_H_12_O_6_	180.18	12	24	1
Fructose	C_6_H_12_O_6_	180.18	12	24	1
Xylose	C_5_H_10_O_5_	150.15	10	20	1
Sucrose	C_12_H_22_O_11_	342.34	23	46	2
Sorbitol	C_6_H_14_O_6_	182.20	12	25	0
Xylitol	C_5_H_12_O_5_	152.17	10	21	0
Glycerol	C_3_H_8_O_3_	92.11	6	13	0
Erythritol	C_4_H_10_O_4_	122.14	8	17	0

**Table 4:** Information index on atomic composition (*I_AC_*), first Zagreb number (*Z*_1_), and global information index (*G*) for the investigated solutes.

Solute	*I_AC_*	*Z*_1_	*G*
Glucose	36.00	58	94.00
Fructose	36.00	60	96.00
Xylose	30.00	48	78.00
Sucrose	67.95	120	187.95
Sorbitol	37.89	50	87.89
Xylitol	31.87	40	71.87
Glycerol	19.79	20	39.79
Erythritol	25.84	30	55.84

## Supplementary Materials

The following are available online at https://www.mdpi.com/article/10.3390/ijms222011044/s1.

## Appendix A

The Kirkwood–Buff (KB) theory of solutions [7,42] describes the effects of solute concentration on the chemical potential of water (*µ_w_*) as follows:

(A1) $${\left(\frac{\partial \mu_{w}}{\partial x_{s}}\right)}_{T,P}=-\frac{RT}{x_{w}\left[1+x_{s}c_{w}\left(G_{ww}+G_{ss}-2G_{ws}\right)\right]},$$

where *T* and *P* are the temperature and pressure, *R* is the universal gas constant, and *c_w_* is the molar concentration of water. The three quantities indicated as *G_ww_*, *G_ss_*, and *G_ws_*, represent the water–water, solute–solute, and water–solute KB integrals, respectively. They are determined by the local fluid structure, i.e., the local density around a given molecule, and can be related to the radial distribution function between the *i* and *j* species, *g_ij_(r)*, as:

(A2) $$G_{ij}=4\pi \int_{0}^{\infty }\left[g_{ij}\left(r\right)-1\right]r^{2}dr,$$

where *r* is the distance between the centers of mass of the two molecules. A positive *G_ij_* value corresponds to an excess of species *j* in the proximity of species *i* over the bulk solution, where the radial distribution function is equal to one; this indicates a favorable interaction between *i* and *j*. In contrast, a negative *G_ij_* value indicates a depletion of species *j* around *i*, and therefore an unfavorable interaction between the two molecules [35].

Equation (A1) can be rewritten in terms of *γ_w_*, the activity coefficient of water, as:

(A3) $${\left(\frac{\partial \ln\gamma_{w}}{\partial x_{s}}\right)}_{T,P}=\frac{x_{s}c_{w}\left(G_{ww}+G_{ss}-2G_{ws}\right)}{x_{w}\left[1+x_{s}c_{w}\left(G_{ww}+G_{ss}-2G_{ws}\right)\right]}.$$

From the Norrish equation, it can be seen that:

(A4) $${\left(\frac{\partial \ln\gamma_{w}}{\partial x_{s}}\right)}_{T,P}=2k_{N}x_{s},$$

and hence:

(A5) $$k_{N}=\frac{c_{w}\left(G_{ww}+G_{ss}-2G_{ws}\right)}{2x_{w}\left[1+x_{s}c_{w}\left(G_{ww}+G_{ss}-2G_{ws}\right)\right]}.$$

At infinite solute dilution, i.e., at *x_s_* → 0, the above equation becomes:

(A6) $$k_{N}=\frac{G_{ww}^{\infty }+G_{ss}^{\infty }-2G_{ws}^{\infty }}{2V_{w}},$$

where *V_w_* is the partial molar volume of water, and all KB integrals are calculated at infinite dilution. Thus, strictly speaking, the Norrish equation is thermodynamically rigorous only at infinite dilution or, more generally, as long as the following condition is satisfied:

(A7) $$\frac{c_{w}\left(G_{ww}+G_{ss}-2G_{ws}\right)}{x_{w}\left[1+x_{s}c_{w}\left(G_{ww}+G_{ss}-2G_{ws}\right)\right]}\approx \frac{G_{ww}^{\infty }+G_{ss}^{\infty }-2G_{ws}^{\infty }}{V_{w}}.$$

In practice, however, the Norrish model can be used with good accuracy over a reasonably wide range of solute concentrations. For example, in aqueous solutions of various nonelectrolytes, including sucrose, the Norrish equation was found to hold up to approximately 60% (*w/w*) of solute [32].
