# Supplementary material for: Water Activity Prediction in Sugar and Polyol Systems Using Theoretical Molecular Descriptors
Source: Int J Mol Sci. 2021 Oct 13;22(20):11044. doi: 10.3390/ijms222011044 (PMC8540113; doi:10.3390/ijms222011044)
Supplement: Supplementary file 1 [file ijms-22-11044-s001.zip › ijms-1406669-supplementary.pdf]

Water Activity Prediction in Sugar and Polyol Systems Using Theoretical Molecular Descriptors

Antonio Zuorro

Supplementary Material

Figure S1. Dependence of the information index on atomic composition (I_{AC}) on the number of hydroxyl groups

Figure S2. Chemical structures of glucose and fructose

Table S1 Water activity data for the system glucose–water

Table S2. Water activity data for the system fructose–water

Table S3. Water activity data for the system xylose–water

Table S4. Water activity data for the system sucrose–water

Table S5. Water activity data for the system sorbitol–water

Table S6. Water activity data for the system xylitol–water

Table S7. Water activity data for the system glycerol–water

Table S8. Water activity data for the system erythritol–water

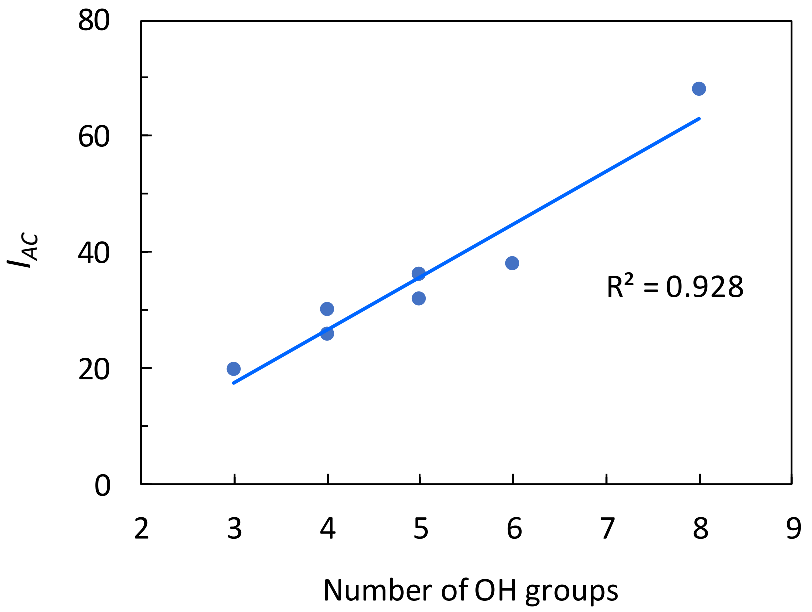

Figure S1. Dependence of the information index on atomic composition (I_{AC}) on the number of hydroxyl groups.

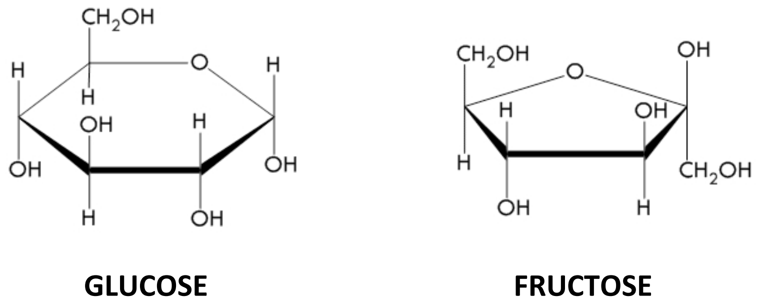

Figure S2. Chemical structures of glucose and fructose.

Table S1. Water activity data for the system glucose–water (a_w is the activity of water, x_s is the mole fraction of the solute and x_w is the mole fraction of water; T = 35 °C) [40].

a_w	x_s	x_w
0.975	0.023	0.977
0.968	0.029	0.971
0.961	0.035	0.965
0.954	0.040	0.960
0.950	0.044	0.956
0.944	0.049	0.951
0.939	0.052	0.948
0.917	0.069	0.931
0.906	0.077	0.923
0.898	0.083	0.917
0.889	0.090	0.910
0.876	0.099	0.901
0.860	0.111	0.889

Table S2. Water activity data for the system fructose–water (a_w is the activity of water, x_s is the mole fraction of the solute and x_w is the mole fraction of water; T = 35 °C) [40].

a_w	xs	x_w
0.971	0.026	0.974
0.963	0.035	0.965
0.942	0.052	0.948
0.929	0.062	0.938
0.922	0.067	0.933
0.913	0.075	0.925
0.890	0.091	0.909
0.882	0.098	0.902
0.873	0.105	0.895
0.861	0.113	0.887
0.851	0.119	0.881
0.836	0.131	0.869
0.819	0.142	0.858

Table S3. Water activity data for the system xylose–water (a_w is the activity of water, x_s is the mole fraction of the solute and x_w is the mole fraction of water; T = 35 °C) [40].

a_w	x_s	x_w
0.980	0.020	0.980
0.978	0.021	0.979
0.974	0.025	0.975
0.971	0.028	0.972
0.967	0.031	0.969
0.966	0.032	0.968
0.963	0.035	0.965
0.960	0.037	0.963
0.955	0.041	0.959
0.951	0.045	0.955
0.947	0.048	0.952
0.943	0.051	0.949
0.939	0.055	0.945
0.934	0.059	0.941

Table S4. Water activity data for the system sucrose–water (a_w is the activity of water, x_s is the mole fraction of the solute and x_w is the mole fraction of water; T = 35 °C) [40].

a_w	x_s	x_w
0.979	0.018	0.982
0.970	0.025	0.975
0.962	0.032	0.968
0.953	0.038	0.962
0.938	0.047	0.953
0.933	0.050	0.950
0.920	0.058	0.942
0.909	0.064	0.936
0.903	0.068	0.932
0.895	0.072	0.928
0.890	0.076	0.924
0.876	0.083	0.917
0.866	0.087	0.913
0.858	0.092	0.908
0.846	0.098	0.902

Table S5. Water activity data for the system sorbitol–water (a_w is the activity of water, x_s is the mole fraction of the solute and x_w is the mole fraction of water; T = 35 °C) [40].

a_w	x_s	x_w
0.980	0.018	0.982
0.971	0.026	0.974
0.961	0.035	0.965
0.955	0.040	0.960
0.949	0.047	0.953
0.943	0.051	0.949
0.933	0.057	0.943
0.927	0.063	0.937
0.920	0.067	0.933
0.916	0.072	0.928
0.901	0.083	0.917
0.895	0.087	0.913
0.888	0.092	0.908
0.878	0.098	0.902
0.872	0.106	0.894
0.862	0.113	0.887

Table S6. Water activity data for the system xylitol–water (a_w is the activity of water, x_s is the mole fraction of the solute and x_w is the mole fraction of water; T = 35 °C) [40].

a_w	x_s	x_w
0.979	0.020	0.980
0.971	0.026	0.974
0.961	0.035	0.965
0.955	0.042	0.958
0.943	0.051	0.949
0.937	0.057	0.943
0.929	0.063	0.937
0.921	0.068	0.932
0.913	0.075	0.925
0.908	0.079	0.921
0.901	0.084	0.916
0.891	0.092	0.908
0.884	0.098	0.902
0.873	0.106	0.894
0.864	0.112	0.888

Table S7. Water activity data for the system glycerol–water (a_w is the activity of water, x_s is the mole fraction of the solute and x_w is the mole fraction of water; T = 35 °C) [41].

a_w	x_s	x_w
0.990	0.010	0.990
0.979	0.021	0.979
0.967	0.033	0.967
0.953	0.046	0.954
0.937	0.061	0.939
0.919	0.077	0.923
0.897	0.095	0.905
0.874	0.115	0.885
0.847	0.137	0.863
0.816	0.163	0.837
0.780	0.192	0.808
0.740	0.225	0.775
0.689	0.265	0.735
0.632	0.312	0.688

Table S8. Water activity data for the system erythritol–water (a_w is the activity of water, x_s is the mole fraction of the solute and x_w is the mole fraction of water; T = 30 °C) [41].

a_w	x_s	x_w
0.993	0.007	0.993
0.985	0.016	0.984
0.975	0.025	0.975
0.964	0.036	0.964
0.956	0.043	0.957
0.940	0.057	0.943
0.926	0.069	0.931
